# Method Development for Assessing Carbamazepine, Caffeine, and Atrazine in Water Sources from the Brazilian Federal District Using UPLC-QTOF/MS

**DOI:** 10.1155/2018/4593793

**Published:** 2018-11-11

**Authors:** Fernando F. Sodré, Cínthia M. P. Cavalcanti

**Affiliations:** ^1^Institute of Chemistry, University of Brasília, Brasília 70910-000, Brazil; ^2^Environmental Sanitation Company of the Federal District (CAESB), Águas Claras 71928-720, Brazil

## Abstract

About 3.0 million people living under a typical tropical savannah climate in the Brazilian Federal District (FD) have faced an unprecedented water crisis. Considering the need for indirect reuse of wastewater for public supply, this work aimed to investigate FD water sources regarding the presence and risks of three contaminants of emerging concern: caffeine, carbamazepine, and atrazine. Samples from two current water sources (Descoberto and Santa-Maria Lakes) and two future water sources of the FD (Paranoá and Corumbá Lakes) were analyzed by solid-phase extraction followed by liquid chromatography coupled to hybrid quadrupole-time-of-flight mass spectrometry (UPLC-QTOF/MS). Method precision and accuracy were satisfactory and limits of quantification ranged from 0.37 to 0.54 ng/L. Higher concentrations were observed for caffeine in the future water sources (39 to 180 ng/L) followed by carbamazepine (5.4 to 25 ng/L) and atrazine (3.9 to 15 ng/L). The less-impacted water sources, in current use in the FD, present caffeine concentrations ranging from 4.8 to 32 ng/L and atrazine levels varying between 2.4 and 5.5 ng/L. Carbamazepine was not detected in these reservoirs. Environmental risk assessment indicates a possible risk for carbamazepine and atrazine, evidencing the need for further studies. No human health risk was depicted within the results.

## 1. Introduction

The capital of Brazil, Brasília, is located in the Brazilian Federal District (FD) under a typical tropical savannah climate with distinct periods of precipitation and humidity. The winter is dry with approximately 120 days without rainfall, resulting in severe problems related to water scarcity and rationing. The most important drinking water systems of the FD (Descoberto Lake and Santa-Maria Lake production systems) have become insufficient to supply about 3.0 million people living in the region. Thus, several actions have already been taken by the Environmental Sanitation Company to improve water availability, such as the use of alternative low-flow water intakes, the constant policing of the water sources, and the minimization of water losses during production processes.

As a result of low rainfall rates for three consecutive years, combined with a lower water recharge and an intense water use, the region is experiencing the largest water crisis in its history. To alleviate this problem, several long-term alternatives were evaluated, two of which were selected for the expansion of the water supply system: the use of Corumbá and Paranoá Lakes as water sources. The former is located beyond the borders of the FD and receives effluents from wastewater treatment plants (WWTPs), either directly or through its tributaries, while Paranoá Lake is an urban water system that receives effluents from two important WWTPs of the FD, as well as urban drainage waters and contaminated waters from tributaries, some of them running through densely populated areas.

Under this new reality, the indirect reuse of water is significant [1] and WWTPs become the most promising sources of water to be recycled [2]. Therefore, monitoring of water quality should incorporate aquatic parameters of emerging concern related to the lifestyle of the modern human societies [3, 4]. In this context, emerging contaminants are of particular interest, as they arise in the environment through a variety of routes [5], may be refractory to conventional treatment methods [6–8], may promote adverse reproductive and developmental effects [9–11], and have been widely used as environmental tracers of several human-related substances [12, 13].

As pointed out by Snyder and Bennoti [14], the implementation of water reuse operations may experience resistance from public opinion mainly due to the presence of emerging contaminants such as pharmaceuticals, personal care products, endocrine disruptor chemicals, and pesticides. Thus, it is necessary to generate reliable information regarding different aspects of this issue, such as the removal efficiency by conventional and advanced drinking and wastewater treatment processes, the impact of wastewater discharge on the presence of such contaminants in drinking water supplies, and the natural attenuation of such contaminants in the environment [14].

Although Descoberto and Santa-Maria Lakes are the main water sources in the FD, most of the work involving the presence of emerging contaminants has been carried out in the waters of Paranoá Lake considering its historical importance and future multiple-use possibilities. Abbt-Braun et al. [15] investigated the presence of pesticides, sweeteners, and perfluorinated substances in Paranoá Lake and observed that high concentrations (nearby WWTPs) decreased through natural processes such as dilution, photolysis, and degradation until reaching the regions near the lake dam where water will be used for public supply purposes. The authors also show that only caffeine and iopromide present concentrations higher than 100 ng/L in the site of the future raw water withdrawal. Da Costa et al [16] investigated the occurrence of emergent contaminants at five sampling points along Paranoá Lake, including the point at the lake dam, where they observed lower concentrations of caffeine, bezafibrate, bisphenol A, diethyl phthalate, and nonylphenol compared to the other points located in the four branches of the lake.

Descoberto Lake was previously investigated for the presence of caffeine, atrazine, atenolol, and DEET, an active ingredient in insect repellents [17]. The concentration of these contaminants varied between 2.6 (DEET) and 10 ng/L (caffeine) being considerably lower than those found in Paranoá Lake.

In Brazil, the monitoring of contaminants in drinking water became mandatory only in 1977, with the publication of BSB Ordinance No. 56/1977, which recommends periodic determinations of 10 inorganic contaminants, 12 pesticides, and 14 organoleptic parameters. After successive revisions over time, water quality standards were gradually increased. Nowadays, determinations of 15 inorganic contaminants, 15 organic substances, 27 pesticides, 7 disinfectants and their by-products, and more than 21 organoleptic parameters are required, through Annex XX of Consolidation Ordinance No. 05/2017, published by the Brazilian Ministry of Health. Within the contaminants investigated in the present work, only atrazine is considered in the Brazilian legislation for raw and treated waters. For the other substances, i.e., caffeine and carbamazepine, there are still no initiatives in Brazil for their inclusion in a monitoring program of national proportions.

This work was motivated by the importance of emerging contaminants in situations of indirect water reuse and by the limited amount of information regarding this class of contaminants in Brazil, especially in the FD. Thus, the present work aimed to develop and apply a method based on the solid-phase extraction of caffeine, atrazine, and carbamazepine followed by the quantification by ultra-efficiency liquid chromatography coupled to a high-resolution hybrid mass spectrometer (quadrupole-time-of-flight). These chemicals were selected due to their use as tracers of anthropogenic discharges in surface waters [18–20].

## 2. Materials and Methods

### 2.1. Chemicals

Methanol and acetone (HPLC grade) were obtained from Scharlau Chemie SA (Spain). Formic acid, acquired from Sigma-Aldrich (USA), was used as mobile phase additive. Ultrapure water was produced in a Milli-Q Academic system (Millipore, USA). Caffeine (98%, CAS 58-08-2) and atrazine (99%, CAS 1912-24-9) were purchased from Fluka Analytical (USA), whereas carbamazepine (99%, CAS 298-46-4) was obtained from Sigma-Aldrich (USA). Stock solutions (200 mg L^−1^), prepared by the solubilization of appropriate amounts of each solid standard in methanol, were kept in amber glass bottles at -10°C. A mixed stock solution containing 400 *μ*g L^−1^ of each standard was used during the optimization of instrumental parameters. For quantification, mixed stock and working solutions were prepared weekly in methanol and kept under refrigerated conditions when not in use. A standard solution (Sciex, Canada) containing a mixture of cesium iodide (CsI, m/z 132.9054) and the synthetic peptide ALILTLVS (m/z 829.5398) was used for mass calibration and tuning.

### 2.2. Study Area and Sampling

**Figure 1:**
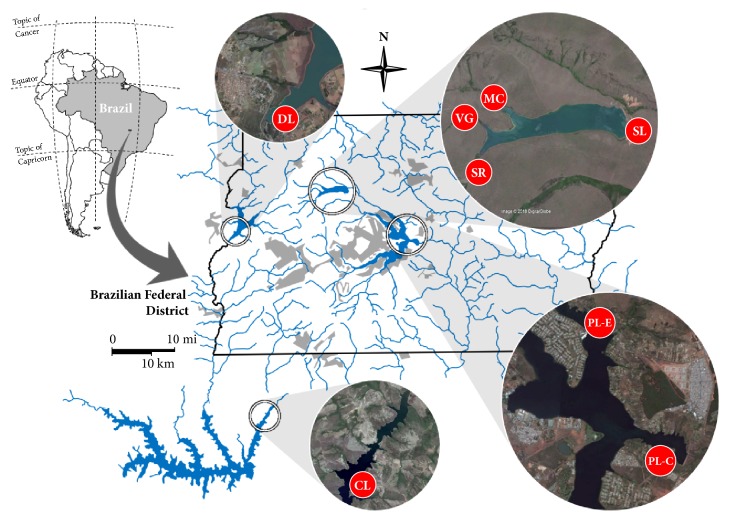
Map showing the Federal District in Brazil and the sampling points selected. DL: Descoberto Lake, SL: Santa-Maria Lake, SR: Santa-Maria River, VG: Vargem-Grande Stream, MC: Milho-Cozido Stream, CL: Corumbá Lake, PL-C: Paranoá Lake (conventional uptake), and PL-E: Paranoá Lake (emergency uptake).

Figure 1 shows the location of the sampling points selected in the present work. The sampling points DL and SL were established at the water intakes of Descoberto and Santa-Maria Lakes, respectively. The water supply systems of both water sources account for approximately 86% of the drinking water production for the population of the FD.

Although located in an environmental protection area, the Descoberto Lake basin suffers from several problems such as invasions of protected areas, high population densities, agricultural activities, and siltation. Santa-Maria Lake is considered the most protected water source of the FD due to its restricted access through the National Park of Brasília. Sampling points were also established in the tributaries of Santa-Maria Lake: Santa-Maria River (SR), Milho-Cozido Stream (MC), and Vargem-Grande Stream (VG).

Two sampling points were established in Paranoá Lake. This urban artificial lake was built in 1959 to generate electric power and to improve the microclimate of the future capital of Brazil, Brasília, but is currently used for recreation, sports, tourism, and fishing. Paranoá Lake also receives urban drainage waters, effluents from two wastewater treatment plants, and other diffuse contributions [15, 21]. The sampling point PL-E is located in the water intake of the emergency water treatment plant (WTP), in operation since October 2017, while the sampling point PL-C is located at the water intake of the conventional WTP that will be in permanent operation in the near future.

Finally, the sampling point CL is located in one of the branches of Corumbá Lake, an artificial lake formed for electric power generation. This lake faces similar problems to those suffered by Descoberto Lake.

A total of 25 samples were collected in different sampling campaigns. Most of them (60%) were from Paranoá Lake. In this case, nine samples were obtained from the conventional water intake point whereas six were obtained at the emergency intake point. Three samples were from Descoberto Lake, three were from Santa-Maria Lake, and one sample was from Corumbá Lake. The remaining three samples were collected in three tributaries of the Santa-Maria Lake.  Table 1 shows details on the samples and sampling periods.

Samples from different depths were collected using a Van Dorn water sampler and transferred to amber glass bottles (1 L). Surface samples were obtained directly into glass bottles. All bottles were previously cleaned in the laboratory and rinsed with the sampled water on site. Samples were transported to the laboratory on ice and preserved at 4^o^C until further preparation steps.

### 2.3. Sample Preparation

In the laboratory, samples were first passed through one or more glass fiber filters (GF-3, 0.7 *μ*m, 47 mm diameter, Macherey-Nagel, Germany) and then through 0.45 *μ*m pore-sized nitrocellulose membranes (47 mm diameter, Scharlau, Spain). Solid-phase extraction (SPE) of the analytes was carried out using a procedure described elsewhere [22]. Briefly, filtered samples (1 L) were passed through 200 mg SPE cartridges (HLB Oasis, Waters, USA) fitted to a lab-made extraction system [23] and to a peristaltic pump (Minipuls Evolution, Gilson, USA). Cartridges were previously conditioned using two aliquots (5 mL) of methanol followed by two aliquots (5 mL) of ultrapure water. Samples were passed through the solid phase at a flow rate of 5 mL min^−1^. After extraction, the cartridges were centrifuged (1 min, 4000 rpm) to remove the excess of water and dried with a gentle flow of N_2_. Analytes were eluted into precleaned glass tubes in a 12-port Prep Sep vacuum manifold (Visiprep DL, Supelco, USA) using two aliquots (3 mL) of methanol followed by another aliquot of a mixture of methanol and acetone (1:1, v/v). The eluates were transferred to evaporation flasks for concentration in a 12-vessel parallel evaporator (Syncore Analyst, Büchi, France) to a final volume of 1.0 mL.

### 2.4. Instrumentation

Analyses were carried out using an Expert Ultra LC100 chromatographic system (Eksigent Technologies, USA) consisting of a binary pump, a vacuum degasser, a thermostated autosampler (LC100-XL), and a thermostated column oven, coupled to a hybrid quadrupole-time-of-flight tandem mass spectrometer (TripleTOF 5600+, Sciex, Canada) with a DuoSpray ion source interface operated in the electrospray ionization (ESI) mode. Nitrogen was produced by a high-purity generator (Genius 3010, Peak Scientific, USA) and used as source gas.

### 2.5. Chromatographic Separation

Separation was performed using a C18 column (Kinetex 2,6 *μ*m EVO, 100 Å, 50 × 4.6 mm) obtained from Phenomenex (USA) at 40°C with gradient elution under a flow rate of 0.80 mL/min. Formic acid solutions (0.1% v/v) prepared in ultrapure water and in methanol were used as mobile phase solvents. The gradient was achieved after 3.5 min by keeping the relative methanol concentration at 15% (initial condition) for 0.5 min, followed by an increase to 50% in 0.5 min, and held constant for 2.5 min. After the elution of analytes, the column was washed with 95% methanol for 1.5 min, readjusted to the initial conditions, and reequilibrated for 2 min. During analyses, the autosampler was kept under 8°C and the injection volume was 2.0 *μ*L.

### 2.6. Mass Spectrometry Conditions

Successive injections of a 100 *μ*g/L caffeine solution were performed to optimize ESI gas parameters in a 2^3^ factorial design. Auxiliary ion source gas 1 (GS1) and ion source gas 2 (GS2) were used as nebulizer and drying gases, respectively, at a back pressure of 60 psi for both parameters. Curtain gas (CUR) was 40 psi. The ESI interface was operated in the positive mode at 650°C with a capillary voltage of 5500 V. Under these conditions the highest sensitivity without in-source fragmentation was observed.

Data acquisition was performed using the high-resolution multiple reaction monitoring (HR-MRM) mode. Firstly, precursor ions were selected by direct infusions of a 0.1% formic acid solution prepared in methanol containing 400 *μ*g/L of each analyte using a declustering potential (DP) of 100 V and a collision energy (CE) of 10 eV. Then, after preliminary direct infusions, product-ions and definitive DP and CE were obtained for each analyte by chromatographic injections of a 100 *μ*g/L mix solution using a 2^2^ factorial design.  Table 2 shows the HR-MRM parameters obtained for each analyte and their chromatographic retention time.

The mass spectrometry system was firstly calibrated using the CsI/ALILTLVS solution under direct infusion. Then, a CMZ solution was used for routine mass tuning on a daily basis. During the analyses, a mix solution was injected every five chromatographic runs for mass calibration. An error up to 2 ppm was considered acceptable. Formic acid was added to all working solutions to favor positive ionization of the target compounds.

### 2.7. Validation

Analytical curves were tested for the homogeneity of variances by the Cochran test. Outliers were verified by the Grubbs test. For the heteroscedastic data, a weighted least squares regression method was performed using weighting factors that produced the lowest sum of the relative errors [24]. Repeatability was based on the analysis of five-point analytical curves performed by the same analyst on the same day. The matrix effect was evaluated for each compound at three concentration levels (5, 95, and 190 *μ*g/L, on-column) using two replicates for each level. It was estimated by comparing the slopes of response curves made in solvent (methanol) and in extracts of a Paranoá Lake sample collected at 1 m depth. Extraction efficiency (in %) was also achieved for another Paranoá Lake sample.

### 2.8. Risk Assessment

The environmental risk was assessed by calculating the risk quotient (RQ_env_) based on the MEC/PNEC ratio, where MEC stands for the measured environmental concentrations obtained in the surface water samples. RQ_env_ was calculated considering the most restrictive Predicted No-Effect Concentration (PNEC) found in the literature for each investigated contaminant. Human health risks were also evaluated by risk quotients (RQ_hum_) comparing the target chemicals concentrations with water quality criteria (WQC) calculated using

(1) $$\begin{array}{c} WQC=\frac{ADI\times P\times BW}{C}, \end{array}$$

where ADI is the acceptable daily intake, in mg/kg, P is the allocation factor considering the percentage of the contaminant ingested via water consumption, BW is the body weight, and C is the daily water consumption. Default values for P (20%), BW (60 kg), and C (2 L) were used considering water consumption for an adult according to the Guidelines for Drinking-Water Quality [25].

## 3. Results and Discussion

### 3.1. Linearity and Limit of Quantification

For all analytes, the five-point analytical curves were heteroscedastic according to the Cochran test. Also, no outliers were depicted using the Grubbs test. The best weighting factor for caffeine and atrazine was 1/*σ*, while for carbamazepine a weighting factor of $1/\sqrt{y}$ provided the lowest sum of the relative errors. Under these conditions, correlation coefficients were significant and higher than 0.99, as can be seen in Table 3.

The limit of quantification (on-column) was admitted as the lowest concentration of the analytical curves for all analytes, i.e., 0.48 *μ*g/L. Under these conditions, signal-to-noise ratios (S/N) were higher than 10.

### 3.2. Precision

Table 4 shows the precision obtained during the construction of weighted analytical curves by external calibration.

Precision was considered satisfactory for all analytes since coefficients of variance below 5% were observed, with the exception of caffeine, where values of 6% were obtained for the highest concentration levels. During the experiments, it was noticed that the standard deviation of the analytical curves was influenced by the ambient temperature, the cleaning and maintenance of the mass spectrometer orifice plate, the stabilization of the analytical signals, and mainly the constant calibration of the exact mass, which must be done frequently throughout the analysis. Thus, results shown in Table 2 were obtained under constant room temperature (20°C), after adequate cleaning of the source and other components of the mass spectrometry system and by periodic injections of the mass calibration solution. The automatic integration of the peaks also influenced the precision, being necessary to check and adjust the baseline manually, mainly for low concentrations. In this sense, higher precision was obtained by parameterizing the noise reduction by 100%.

### 3.3. Accuracy

**Figure 2:**
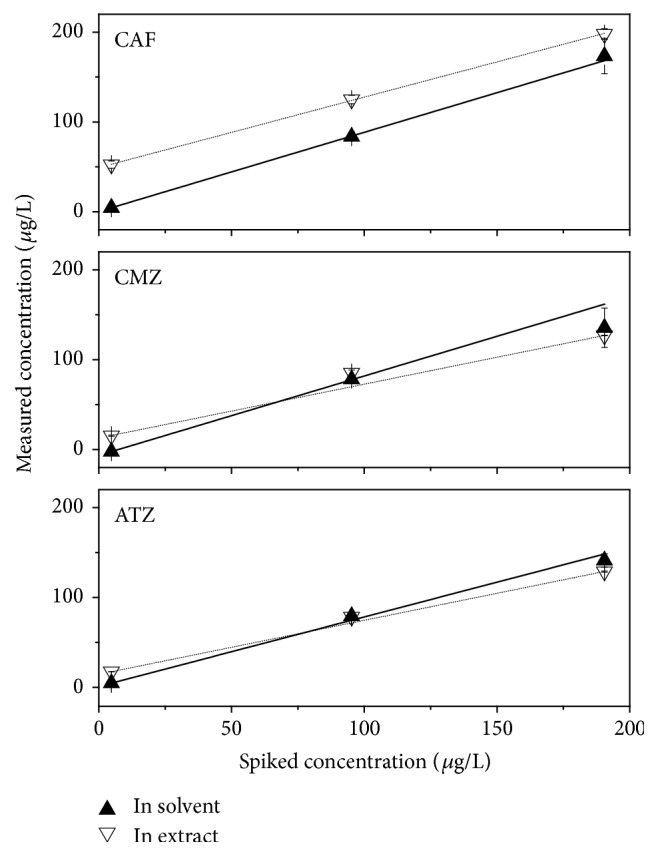
Matrix effects on the analytical response of the investigated contaminants.

The matrix effect (ME) was evaluated by plotting two curves: the first one obtained by the analysis of three solutions containing increasing concentrations of the analytes in methanol and the second one with the same concentrations of the analytes in a sample matrix, i.e., an extract of a Paranoá Lake sample collected at 1 m depth. Both curves were plotted with three concentration levels due to the small amount of sample extract available.  Figure 2 portrays linear correlations obtained during ME experiments.

For all analytes, the matrix effect was manifested in order to attenuate the analyte response with a tendency to underestimate higher concentrations. Caffeine suffered less influence of the matrix (14% attenuation), followed by atrazine (18%) and carbamazepine (19%). The slopes were statically compared using Student's* t*-test based on the standard errors of the regressions [26]. Experimental* t*-values for caffeine, carbamazepine, and atrazine were 3.713, 1.113, and 2.048, respectively, being below the critical value of 4.303 (95% confidence interval) and indicating that the matrix effect was not significative. For caffeine, more intense responses were observed for the in-extract curve in comparison to the in-solvent one.  Table 5 shows that there was satisfactory recovery for caffeine at the three concentrations investigated. Satisfactory recovery rates at high concentrations (190 ng/L) are important for caffeine, considering that higher levels of this substance are expected in Brazilian natural waters compared to the other tested analytes [17, 27, 28].

It is observed in Figure 2 that the central points of the curves in solvent and in extract for carbamazepine and atrazine were similar, whereas maximum and minimum concentrations differ leading to the attenuation of the sensitivity in the curves plotted in extract. Considering only the lowest concentrations, i.e., compatible with the environmental levels commonly found in the region [15], a 13% attenuation was estimated for carbamazepine. For atrazine, there was no change in attenuation at the lower concentration levels. However, for both analytes Table 5 shows more satisfactory recoveries for the lower concentration levels, making the results achieved also satisfactory.

Accuracy was also assessed by a recovery test for extraction efficiency. In this case, a sample of Paranoá Lake was enriched with 55 ng/L of each analyte and submitted to extraction and analysis using the weighted analytical curves described in Table 4.  Table 5 shows satisfactory recoveries ranging from 78±5 to 112±7%.

### 3.4. Emerging Contaminants in Water Sources

Method limits of quantification (LOQm) were expressed by the instrument LOQ (Table 3) multiplied by the extraction recovery (Table 5) and divided by the preconcentration factor of 1000 times. Values for caffeine, carbamazepine, and atrazine were 0.49, 0.37, and 0.54 ng/L, respectively. Prior to analysis, extraction blank controls, obtained by the analysis of 1 L of ultrapure water, in triplicate, revealed the presence of 4.4 ng/L of caffeine. As solvent blank controls did not reveal the presence of any analyte, this interfering concentration was considered in the calculation of the final concentrations of caffeine in the samples.

Table 6 shows the concentrations of caffeine, carbamazepine, and atrazine in the water sources in current use in the FD.

Only caffeine and atrazine were detected in Descoberto and Santa-Maria Lakes in all samples investigated. The concentration of the former was higher varying between 10 and 32 ng/L in Descoberto Lake and from 4.8 to 10 ng/L in Santa-Maria Lake.

Higher levels of caffeine in the Descoberto Lake are expected due to the occupation of adjacent areas by condominiums and by the increasing population density observed in the region in the last years. These factors may contribute to the presence of caffeine, a known tracer of human activities [20, 29, 30]. However, the presence of caffeine in Santa-Maria Lake was not expected, even under lower concentrations, since this compartment is under restricted access within the borders of the National Park of Brasília. In view of these results, we sought to investigate samples from three tributaries from this reservoir in an attempt to trace possible sources of contamination. Results revealed significantly higher concentrations (75 to 123 ng/L) of caffeine in these streams (Table 6) compared to those determined in Santa-Maria Lake. Therefore, there is evidence of potentially contaminating human activities in the drainage areas of the tributaries. The possible causes of the presence of caffeine in these streams have not been identified yet, but high levels of coliforms in these tributaries (data not shown) suggest a common source for chemical and biological contamination.

Atrazine levels varied between 2.4 and 5.5 ng/L in both compartments and probably arise due to minor diffuse sources related to agricultural activities in the surrounding areas. No significant differences were observed when results from different seasons were compared indicating that pollution processes may be stable over the year.

Table 7 shows the levels of the emerging contaminants investigated in the future sources of water in the FD.

The three analytes investigated were found in all samples of the Corumbá and Paranoá Lakes. Concentrations of caffeine were higher in both lakes, followed by carbamazepine and atrazine. As expected, concentrations were also consistently higher in these reservoirs compared to the current water sources of the FD. The presence of contaminants in samples from different depths indicates the vertical mixture of the waters of the Paranoá Lake. However, no further tendency was depicted within the samples investigated.

No significant differences were observed in the results considering both sampling points of Paranoá Lake. Caffeine Levels varied between 39 and 180 ng/L in Paranoá Lake considering all investigated samples, corroborating previous reports regarding such contaminant in the lake. In the sampling point PL-C, Abbt-Braun et al. [31] report caffeine concentrations varying from 28 to 193 ng/L during sampling campaigns carried out in 2010. Our results, from samples collected in 2017, show that the sources of contamination remained stable over the last few years in the surroundings of the lake. In the emergency point (PL-E), previous studies revealed caffeine levels varying between 29 and 138 ng/L [16, 31]. In Corumbá Lake, a similar concentration was depicted in comparison with the results portrayed for Paranoá Lake, suggesting similar degrees of anthropic influence in both reservoirs.

Carbamazepine concentrations ranged from 5.4 to 25 ng/L in the samples from Paranoá Lake. For Corumbá Lake, a concentration of 8.5 ng/L was obtained. Again, no significative differences were observed between sampling points, seasons, and depths investigated. A previous report also found carbamazepine in both sampling points of Paranoá Lake in concentrations varying from <5 to 16 ng/L. Atrazine levels, varying from 3.9 to 15 ng/L, were also similar to previous results corroborating with the scenario of contamination that has remained stable since 2010 in the FD.

### 3.5. Risk Assessment

**Figure 3:**
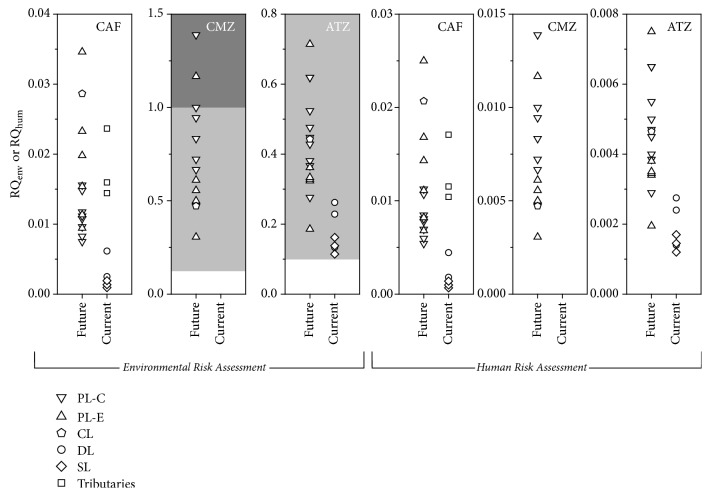
Environmental and human risk assessments of selected emerging contaminants in future and current water sources of the Brazilian Federal District. Dark and light grey regions indicate risk and possible risk, respectively. Blank regions indicate no risk.

Figure 3 portrays risk quotients for environmental and human risk assessment considering the presence of caffeine, carbamazepine, and atrazine in the water sources of the FD.

For the environmental risk assessment, risk quotients (RQ_env_) were calculated as suggested by the Technical Guidance Document on Risk Assessment of the European Commission [32], where the measured environmental concentrations (MEC), portrayed in Tables 6 and 7, are evaluated against previously reported PNEC values. The PNEC can be described as the concentration limit at which harmful effects on organisms will most likely not occur. For aquatic systems, a PNEC should be derived that, if not exceeded, ensures an overall protection of the environment. In the present work, the most restrictive PNECs, representing the worst-case scenario, were selected in literature for carbamazepine (18 ng/L) [33], atrazine (21 ng/L) [34], and caffeine (5200 ng/L) [35].

As PNEC is an estimate, a restrictive demarcation of what is “acceptable” or “not acceptable” is not possible for MEC values below or above this parameter, respectively. Therefore, for a more realistic risk assessment, it is considered that RQ greater than 1 may imply risk while values lower than 0.1 indicate no risk. Intermediate values indicate possible risk as well as the need for further studies [28, 35]. Figure 3 shows no ecological risks for caffeine in both current and future water sources of the FD. Higher RQ_env_ is noticed for carbamazepine, with three samples from Paranoá Lake presenting environmental risk. The remaining samples are classified in a situation of possible risks. Considering that carbamazepine was not detected in the current water sources of the FD, no risk was depicted. For atrazine, all samples investigated in the present work were in the range of possible risks.

For human risk assessment, it was also considered a worst-case scenario where removal efficiency during drinking water treatment processes was null. Water quality criteria were derived considering ADI data available in the literature for carbamazepine (300 ng/kg) [6, 36] and caffeine (1200 ng/kg) [37]. Using (1), these values provide WQCs of 1800 and 7200 ng/L for carbamazepine and caffeine, respectively. For atrazine, a WQC of 2000 ng/L, corresponding to the drinking water standard proposed by the Brazilian Ministry of Health [38], was considered. Human risk quotients (RQ_hum_) portrayed in Figure 3 were based on MEC/WQC ratios and indicate no risk for all analytes investigated in this work, to the best of our knowledge.

## 4. Conclusions

A method based on the solid-phase extraction followed by quantification using liquid chromatography coupled to high-resolution hybrid mass spectrometry (UPLC-QTOF/MS) was developed and applied for the quantification of caffeine, carbamazepine, and atrazine in water sources of the Brazilian Federal District.

Accuracy was considered satisfactory considering matrix effects, as well as recoveries experiments, carried out with samples collected in Paranoá Lake. Precision was also satisfactory under weighted least squares regressions using the most appropriated weighting factors.

Concentrations of the investigated analytes were consistently higher in the future water sources as they receive urban drainage waters, effluents from wastewater treatment plants, and other diffuse contributions. As a result, possible environmental risks were depicted for carbamazepine in the future water sources. Atrazine levels in all water sources were also in a range of possible environmental risks. No risk for human health was estimated based on the worst-case scenario where removal in water treatment plants is not achieved.

Our results point towards a crucial role of indirect water reuse in situations of water scarcity and rationing. Receiving waters may contain several contaminants of recent concern that should be investigated to ensure the safe use of water for different purposes. Although risks to human health have not been evidenced in this work, our results may be useful for constructing a reliable contamination scenario to other alternative and more complete risk assessment models.

## Figures and Tables

**Table 1:** Characteristics of the investigated samples and acronyms used in the present work.

Aquatic system	Acronym	Coordinates	Sampling depth (m)	Season
Paranoá Lake (Conventional)	PL-C	15°47'36.9"S 47°47'22.9"W	1, 5 and 10	Rainy and dry
Paranoá Lake (Emergency)	PL-E	15°44'37.2"S 47°49'51.9"W	1, 5 and 10	Rainy
Corumbá Lake	CL	16°12'26.7"S 48°09'55.2"W	0	Dry
Descoberto Lake	DL	15°46'41.5"S 48°13'52.9"W	9 and 16	Rainy (only 16 m) and dry
Santa-Maria Lake	SL	15°40'33.2"S 47°57'19.6"W	9 and 16	Rainy (only 16 m) and dry
Santa-Maria River	SR	15°40'58.1"S 48°01'09.8"W	0	Dry
Vargem-Grande Stream	VG	15°40'23.4"S 48°01'11.3"W	0	Dry
Milho-Cozido Stream	MC	15°40'11.8"S 48°00'24.0"W	0	Dry

**Table 2:** Acquisition parameters used in the UPLC-QTO/MS system.

Analyte	Formula	Exact mass (Da)	Precursor ion (Da)	Product-ion (Da)	DP (V)	CE (eV)	RT (min)
CAF	C_8_H_10_N_4_O_2_	194.08037	195.0877	138.0662^a^	100	25	1.74
				195.0877			
				110.0349			
CMZ	‎C_15_H_12_N_2_O	236.094963	237.1022	194.0964^a^	70	30	2.27
				237.1022			
				192.0808			
ATZ	C_8_H_14_ClN_5_	215.093773	216.1010	174.0541^a^	100	30	2.55
				216.1010			
				104.0010			

^a^Transitions used for quantification. DP: declustering potential, CE: collision energy, EP: entrance potential, CEP: collision cell entrance potential, CXP: collision cell exit potential, RT: retention time.

**Table 3:** Work range and linearity of the external calibration curves.

Analyte	Work range (*μ*g/L)	Weight	R^2^	LOQ (*μ*g/L)	S/N at LOQ
CAF	0.48 to 300	1/*σ*	0,99	0.48	13.7
CMZ	0.48 to 300	$1/\sqrt{y}$	0,99	0.48	20.6
ATZ	0.48 to 300	1/*σ*	0,99	0.48	16.6

**Table 4:** Coefficients of variance for intraday analysis of mixed solutions of the analytes.

Concentration^a^	Precision (%)^b^		
	Atrazine	Caffeine	Carbamazepine
0.48	1	5	3
2.4	4	2	4
12	3	5	2
60	2	6	5
300	1	6	5

^a^Concentrations in column (*μ*g/L) used the for the construction of analytical curves.  ^b^Peak areas for six replicates

**Table 5:** Percentage of recovery obtained for spiked extracts and for a Paranoá Lake sample spiked with all analytes.

Samples	Recovery (%)		
	Caffeine	Carbamazepine	Atrazine
Extract^a^ spiked with 5.0 *μ*g/L	83±11	79±9	107±6
Extract^a^ spiked with 95 *μ*g/L	81±8	77±9	74±7
Extract^a^ spiked with 190 *μ*g/L	80±8	62±8	64±6
Natural water^b^ spiked with 55 ng/L	102±6	78±5	112±7

^b^Extracts of a Paranoá Lake sample (1 m) obtained after solid phase extraction.  ^a^Filtered (0.45 um) Paranoá Lake sample.

**Table 6:** Concentrations of caffeine, carbamazepine, and atrazine in the current water sources of the Brazilian Federal District and in selected tributaries.

Analytes	Concentration (ng/L)						
	DL		SL		SR	VG	MC
	*9 m*	*16 m*	*9 m*	*16 m*	*0 m*	*0 m*	*0 m*
CAF	13 (D)	32 (R) 10 (D)	4.8 (D)	10 (R) 7.0 (D)	83 (D)	75 (D)	123 (D)
CMZ	ND (D)	ND (R) ND (D)	ND (D)	ND (R) ND (D)	ND (D)	ND (D)	ND (D)
ATZ	5.5 (D)	2.8 (R) 4.8 (D)	3.4 (D)	2.4 (R) 2.9 (D)	ND (D)	ND (D)	ND (D)

CAF: Caffeine, CMZ: Carbamazepine, ATZ: Atrazine, DL: Descoberto Lake, SL: Santa-Maria Lake, SR: Santa-Maria River, VG: Vargem-Grande Stream, MC: Milho-Cozido Stream, ND: Not detected, R: Rainy season, D: Dry season

**Table 7:** Concentrations of caffeine, carbamazepine, and atrazine in the future water sources of the Brazilian Federal District.

Analyte	Concentration (ng/L)						
	CL	PL-C			PL-E		
	*0 m*	*1 m*	*5 m*	*10 m*	*1 m*	*5 m*	*10 m*
CAF	149 (D)	77 (R) 81 (R) 58 (D)	77 (R) 61 (R) 43 (D)	50 (R) 39 (R) 56 (D)	49 (R) 103 (R)	121 (R) 80 (R)	180 (R) 59 (R)
CMZ	8.5 (D)	17 (R) 15 (R) 15 (D)	25 (R) 15 (R) 18 (D)	15 (R) 13 (R) 12 (D)	5.4 (R) 8.9 (R)	21 (R) 11 (R)	9.0 (R) 10 (R)
ATZ	9.3 (D)	9.4 (R) 13 (R) 10 (D)	13 (R) 11 (R) 9.0 (D)	5.8 (R) 7.7 (R) 8.0 (D)	3.9 (R) 6.9 (R)	15 (R) 7.0 (R)	7.6 (R) 6.8 (R)

CAF: Caffeine, CMZ: Carbamazepine, ATZ: Atrazine, CL: Corumbá Lake, PL-C: Paranoá Lake (Conventional uptake), PL-E: Paranoá Lake (Emergency uptake), R: Rainy season, D: Dry season

## Data Availability

The data used to support the findings of this study are available from the corresponding author upon request.
